# The Effect of Soy and Whey Protein Supplementation on Glucose Homeostasis in Healthy Normal Weight Asian Indians

**DOI:** 10.1155/2023/2622057

**Published:** 2023-07-10

**Authors:** Sucharita Sambashivaiah, Mark Cope, Ratna Mukherjea, Sumithra Selvam, Nivya George, Rebecca Kuriyan, Anura V. Kurpad

**Affiliations:** ^1^Department of Physiology, St John's Medical College, Bengaluru, India; ^2^International Flavors & Fragrances Inc., St Louis, MO, USA; ^3^Division of Epidemiology, Biostatistics and Population Health, St John's Research Institute, Bengaluru, India; ^4^Currently-Institute of Human Virology, University of Maryland School of Medicine, Baltimore, MD, USA; ^5^Division of Nutrition, Department of Physiology, St John's Medical College, St John's Research Institute, Bengaluru, India

## Abstract

Milk and legumes are good source of protein foods used to sustain muscle mass, but their effects on postprandial glucose homeostasis and energy metabolism may be different. This is relevant, for example, in the dietetic response to obesity or diabetes, where the intake of high-quality protein is often increased significantly. The objective of this study was to characterize the acute effect of whey and soy protein (15% vs. 30%) on glucose homeostasis, energy metabolism, and satiety. Healthy, normal body mass index (BMI) Indian adult males aged 20–35 years (*n* = 15) received 4 test meals (2 proteins (soy vs. whey) and 2 doses (15% vs. 30% protein: energy ratio)). Blood samples were collected serially after the meal to calculate the incremental area under the curve for plasma glucose and insulin. Energy expenditure and substrate oxidation were measured after the meal. Satiety was measured with a visual analogue scale. The insulin response, represented by the incremental area under the curve, was significantly higher for the 30% whey compared to the 30% soy protein meal (*p* < 0.01) but was not significantly different between the 15% protein doses. There were no differences in the plasma glucose response across protein sources or doses. The mean peak fat and carbohydrate oxidation, satiety, and energy expenditure did not differ between the protein sources and doses. In conclusion, at higher doses, whey protein has a greater insulinogenic response, compared to soy protein, and exhibits a dose-response effect. However, at lower doses, whey and soy protein elicit similar insulinogenic responses, making them equally effective protein sources in relation to glucose homoeostasis.

## 1. Introduction

The impact of dietary protein intake on glucose homeostasis through its action on insulin secretion has gained attention especially among Asian Indians due to growing burden of type 2 diabetes (T2D) [1]. Even before changes among T2D are explored, it is imperative to understand the impact of protein intake among healthy individuals. This is because nonpharmacological strategies are important in the prevention and management of T2D, and data from healthy population could form the foundation for the same. The role of postprandial hyperglycemia and its consequences have been of interest in understanding the pathophysiology of T2D. It has been established that, during the postprandial phase, a rapid increase in blood glucose levels, including hyperglycemic spikes, occurs in those with T2D [2]. This may be relevant as postprandial hyperglycemia has been linked to complications.

Nutrient preload, by manipulating the sequence of macronutrient ingestion during the meal, is one of the novel nutritional approaches that have proven effective in reducing postprandial hyperglycemia [3]. The beneficial effect of noncarbohydrate nutrient preloads includes their ability to promote insulin secretion [4]. However, the mechanisms driving hyperinsulinemia are not clear. For instance, this could be linked to specific amino acid concentrations in protein preloads [5]. In an Asian Indian context, the consumption of quality protein is poor [6]. Two common sources of protein consumed among Asian Indians are milk and legumes [7, 8]. The acute postprandial glycemic and insulinogenic effect of milk (whey) protein is different from legume (soy) protein [9]. This has been attributed to the differing proportions of branched-chain amino acid content of these proteins. However, the consumption of milk/dairy products and resultant hyperinsulinemia has been suggested to produce less than desirable long-term effects in healthy individuals, including insulin resistance [10]. Thus, as a start, there is a need to understand the effects of doses of specific protein intakes on glucose homeostasis in healthy Asian Indians. Currently, there are no data available regarding the type (milk vs. legume) and the dose of protein intake on glucose homeostasis among Asian Indians. Therefore, the primary aim of the present study was to evaluate the acute effects of two proteins, whey protein (WP) and isolated soy protein (ISP), and two doses of each (15 and 30% of energy) on glucose homeostasis. The secondary aim of the study was to explore the changes in energy metabolism and satiety following WP and ISP (15 and 30% of energy) among healthy Asian Indians.

## 2. Materials and Methods

### 2.1. Subject Recruitment

Healthy adult males between the ages of 20–35 years were recruited from in and around St. John's Medical College and Hospital, Bengaluru, India. All participants were screened for inclusion and exclusion criteria. Participants were excluded if they were underweight (BMI <18.5 kg/m^2^), had a history of acute weight loss, and diagnosed with T2D, hypertension, hypercholesterolemia, hyperbilirubinemia, anaemia, cancer, lactose intolerance, protein, or any food allergy, or if they were on any medication or medical condition which could affect the selected outcome measures. In total, 23 participants were screened, out of which 7 participants were excluded due to the exclusion criteria. Of the total 16 participants who were recruited and randomized, 15 participants completed all 4 experiments: 1 subject dropped out after 2 experimental days due to an acute medical condition (dengue fever) not related to the study. The study obtained ethical approval from St John's National Academy of Health Sciences Institutional Ethics Committee on January 23, 2015. The IEC study reference number is 156/2014 (clinical trial registration number CTRI/2018/03/012426). The experimental protocol was explained to all the participants, and their written informed consent was obtained.

### 2.2. Sample Size Estimation

The sample size was estimated based on hyperinsulinemic response to 50% soy and whey protein [11]. The comparison of AUC of insulin between soy and whey protein was considered as the primary outcome for estimating the sample size. To observe a minimum difference of 7.5 nmol/120 min between 30% soy and whey protein with the standard deviation of 5 nmol, 80% power and 1% level of significance (Bonferroni adjustment for multiple comparisons within cross overtrial) were required, and the sample size required was 12. The current study was able to achieve a sample size of *n* = 15.

### 2.3. Method of Randomization and Concealment

The computer-generated randomization sequence for the order of intervention and sequence allocation was generated by an independent statistician. The order of intervention for each participant (30% whey, 30% soy, 15% whey, and 15% soy protein) was randomly assigned (a sequence number that is 1 to 4). In total, 16 participants were randomly allocated into 4 treatment sequence using block randomization (a block size of 4). A copy of all randomization lists was maintained in a sealed envelope with an independent authority in the research institute. Investigators were blinded, and the random allocation of the study participants was carried out by an independent person.

### 2.4. Questionnaire and Body Composition

Each participant underwent detailed dietary history, clinical, and anthropometric examination. Body fat and lean mass were measured by using dual X-ray absorptiometry (DXA). Whole-body and regional body compositions were estimated using the Lunar Prodigy Advanced PA+301969 (GE Medical Systems, USA) whole-body scanner, with software version 12.30. DXA scans were performed with the subject wearing light clothing and no metal objects, by the same laboratory technician. The mass of the lean soft tissue, fat, and bone mineral for the whole body and specific regions were obtained [12].

### 2.5. Experimental Details

Participants reported to the metabolic ward in the evening prior to testing. All participants received a standard evening meal, calculated as a quarter of the daily energy requirement. The composition of dinner was constant for all the participants and was served at the metabolic kitchen in the division of nutrition, following which all the participants slept in the metabolic ward for at least 8 hours. The participants were woken up at 5 am, and the first voided urine sample was collected. They were taken to the adjoining metabolic laboratory, intravenously cannulated and rested for 30 minutes. Basal blood samples were collected, following which their resting energy expenditure and substrate oxidation were measured by indirect calorimetry and visual analogue scales (VAS), for appetite was administered before the consumption of the test meal. Physical activity level (PAL) were calculated based on a physical activity questionnaire [13].

**Figure 1:**
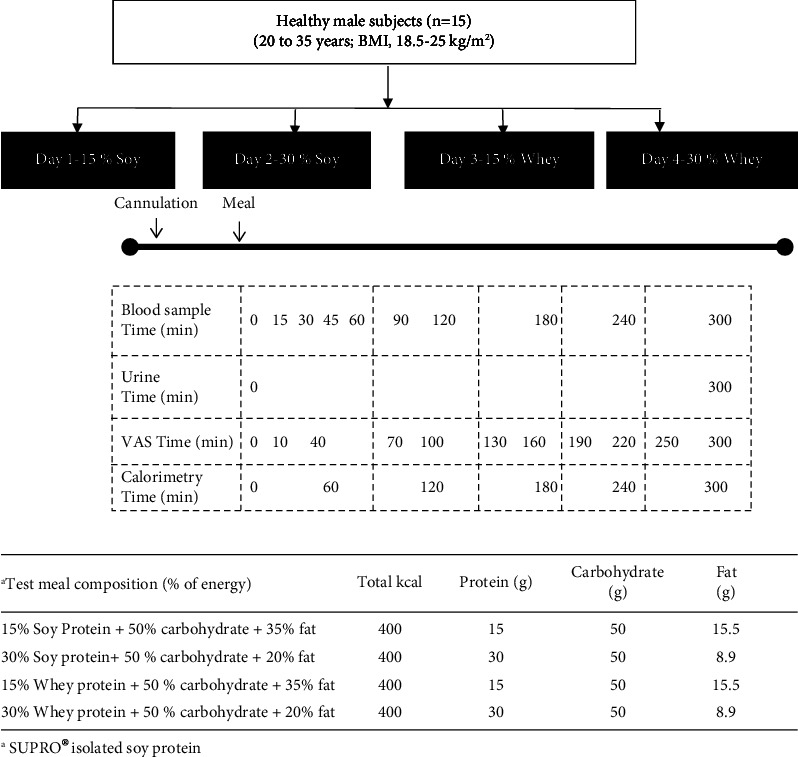
Experimental protocol (a) and energy composition of the test meal (b).

The test meal was vanilla flavored and weighed 85 g with an energy content of 400 kcal. The test meal was a liquid meal with macronutrient content (15% soy, 30% whey protein, 50% carbohydrate, and 20% and 30% fat (Figure 1)), and gastric emptying time could play an impact on the hormonal and satiety responses [14–16]. The test meal protein consisted of either isolated soy (SUPRO® isolated soy protein) or whey protein in two doses each. The meals were matched for all components, but the only varying components were the protein sources. The carbohydrate source was predominantly sugar with a small amount of maltodextrin. The fat source was high oleic sunflower oil.

Each participant randomly received 4 test meals on different days, separated by at least 3 days of washout. The protein type (soy and whey) and dose (15% and 30%) of the test meals are presented in Figure 1. The test meals were prepared by adding the preprepared meal powder to 300 ml water at room temperature and making sure it was homogenously distributed. The test meals were consumed in 5–10 minutes, and the entire consumption was ensured by weighing the containers before and after consumption. All participants could complete their test meals. The study measurements continued for 5 hours postprandially. Details of the study protocol are represented in Figure 1. Blood samples were collected at 15, 30, 45, 60, 90, 120, 180, 240, and 300 min after the test meal. Whole blood glucose was estimated immediately (described below). For plasma insulin measurements, blood was collected in heparinized tubes, cold-centrifuged, and plasma stored at −80°C until analysis. The urine sample was collected at the end of the test meal for urinary nitrogen analysis. A minimum window of 3 days was maintained between the experiments. All 4 experiments were completed within 21 days.

#### 2.5.1. Visual Analogue Scales

Individual subjective indices of appetite were recorded for the duration of the experiment. Four 100 mm visual analogue scales for hunger, thoughts of food, urge to eat, and fullness of stomach were administered. The scale was administered each time in triplicate, and a mean of 3 readings were expressed as percentage of scale [17]. The time points during which VAS was administered have been indicated in Figure 1.

#### 2.5.2. Biochemistry

Plasma glucose measurements were performed by the glucose oxidase method on a bedside glucose analyzer (GM9D, Analox Instruments, London, UK). The intraassay coefficient of variation for this method (using 144.1 mg dL-1 (8 mmol L-1) standards) was <1%, while the interassay coefficient of variation has been <5%. Blood samples for insulin measurements were analyzed by electrochemiluminescence (Elecsys 2010, Roche Diagnostics, Manheim, Germany). The intraassay CV for insulin was 3%, and the interassay CV was 1.3%. The urinary nitrogen was analyzed by the micro-Kjeldahl method [18].

#### 2.5.3. Calorimetry

Calorimetry was performed using a ventilated hood by using an open-circuit calorimeter. Flow calibration was undertaken by burning a known quantity of 99% pure alcohol and measuring the total O_2_ consumption and CO_2_ production. The measurement of minute-to-minute oxygen consumption (VO_2_) and carbon dioxide (VCO_2_) production was made at the baseline and at the end of every hour (last 15 minutes) for 5 hours after the experimental meal. The respiratory quotient (RQ) was calculated as the ratio of VCO_2_ to VO_2_. The resting energy expenditure (EE) was calculated by the Weir formula [19]. Substrate oxidation was calculated from the gas exchange values using stoichiometric equations [20]. In brief, the nonprotein RQ was calculated from gas exchange corrected for protein oxidation based on the urinary nitrogen excretion at the baseline and following the protein meal by timed urine collections. The nonprotein RQ was used to calculate fat and carbohydrate oxidation. These rates were examined every hour (g/min) and compared between the WP (15% vs. 30%) and ISP (15% vs. 30%).

### 2.6. Statistical Analyses

This was a double-blind randomized trial, with randomization codes generated by an independent statistician. Baseline characteristics were reported using mean and standard deviation. The outliers were detected using box plots and the generalized extreme studentized deviate test and were removed from statistical analysis. Assumption of normality was checked using the Kolmogrov–Smirnov test and the Q-Q plot. Non-normal data were log transformed. The mean and the standard error of mean for plasma glucose and insulin were plotted over time. The incremental area under the curve (iAUC) was derived for plasma glucose, insulin response, VO_2_, VCO_2_, RQ, and EE for each of the interventions. The peak response for calorimetry measures, % fat, and CHO oxidation over 5 hour was considered for the analysis. Each outcome was compared between the four interventions using repeated measures analysis of variance (RMANOVA). Post hoc Bonferroni analysis was performed only when the *F* test showed statistical significance. *p* values less than 5% were considered statistically significant. All the analyses were performed using SPSS version 23.0 (IBM SPSS Statistics for Windows, version 22.0. Armonk, NY: IBM Corp).

## 3. Results

**Figure 2:**
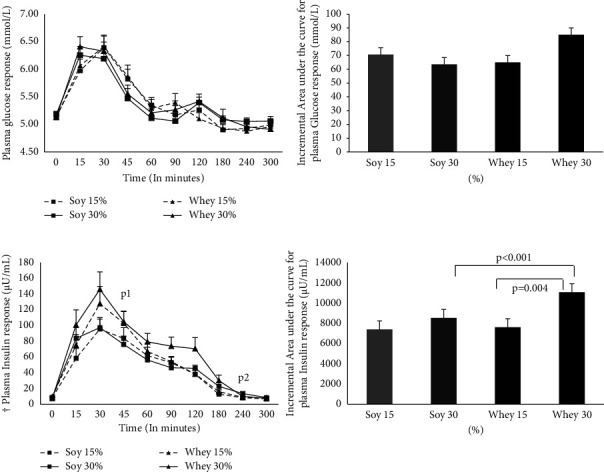
Plasma glucose (mmol/L) (a) and plasma insulin (*μ*U/mL) (b) response to whey and soy protein (15% & 30%). Data represented as mean ± SEM. ^†^Comparison of iAUC between the four meals using RMANOVA; *p* < 0.001. p1: comparison of plasma insulin at 30 minutes from baseline in each intervention, *p* < 0.01; p2: comparison of plasma insulin at 240 minutes from baseline in each intervention, *p* > 0.05.

Baseline characteristics of the study participants are given in Table 1. Plasma glucose and plasma insulin responses to various test meals (mean ± SE of mean) are illustrated in Figure 2. There was a significant change in the mean plasma insulin (*μ*U/mL) in each of the test meals over time. The mean plasma insulin peaked at 30 minutes in all the four interventions and reached the baseline at 240 minutes, nonsignificantly from the baseline. The mean iAUC for plasma insulin was significantly different between the four test meals (*p* < 0.001), i.e., 7384, 8532, 7609, and 11057 for ISP 15%, ISP 30%, WP 15%, and WP 30%, respectively. The post hoc test showed that the mean iAUC for plasma insulin after the ingestion of 15% WP was significantly lower than 30% WP (*p* = 0.004), 30% ISP was significantly lower than 30% WP (*p* < 0.001), and the mean iAUC for plasma insulin for 15% ISP was also significantly different from the 30% WP iAUC. However, the mean insulin of 15% ISP was not significantly different from that of 15% WP and 30% ISP. The mean iAUC for plasma glucose (mmol/L) was 70.71, 63.53, 65.01, and 85.06 for ISP 15%, ISP 30%, WP 15%, and WP 30%, respectively. There was no significant difference in the mean iAUC of plasma glucose levels between the test meals (*p* = 0.11). The peak response and iAUC for the calorimetry measure of VO_2_, VCO_2_, RQ, EE, and VAS scores are presented in Table 2. There was no significant difference in the mean iAUC values of calorimetry measures, VCO_2_ (L/min), RQ, and VAS score between the four interventions except for VO_2_ (*p* = 0.04) and EE (*p* = 0.06). The mean iAUC for VO_2_ (L/min) of 30% WP was significantly higher than that of 15% ISP. The mean iAUC of EE was noted to be higher in 30% WP than that in 30% ISI and 15% WP.

While comparing the peak responses, the mean peak response VO_2_ (L/min) was significantly higher for 30% WP than that for 15% WP. For EE measure, 30% WP was significantly higher than 15% WP meal; similarly, 30% soy was significantly higher than 15% soy. None of the other measures were significantly different between the test meals. Data on calorimetry measures, VO_2_, VCO_2_, RQ, and EE over the 5-hour measurement period are presented in Supplementary Figure 1. There was no significant interaction (time × group effect) noted when calorimetry measures over the 5-hour measurement period were compared between the test meals except for EE (*p*=0.035). At the baseline, there was no significant difference in % fat and CHO oxidation across the four meals. Peak decrement response in % fat oxidation and increment in CHO oxidation over 5 hr was analyzed. The mean peak decrements in % fat oxidation were 11.91, 11.63, 13.15, and 22.29 for ISP 15%, ISP 30%, WP 15%, and WP 30%, respectively. The mean peak increments in % CHO oxidation were 88.09, 88.36, 86.83, and 77.70 for ISP 15%, ISP 30%, WP 15%, and WP 30%, respectively. The mean peak % fat and CHO oxidation values were not significantly different between the four test meals.

## 4. Discussion

The current study demonstrated that insulinogenic response was significantly higher for 30% WP than that for 30% ISP among healthy normal weight Asian Indians. At a lower dose (15%), WP and ISP elicited similar insulinogenic responses among normal weight healthy Asian Indians. There was no difference in plasma glucose across protein sources or doses. The mean peak % fat, CHO oxidation, satiety, and energy expenditure did not differ between protein source and doses.

The habitual protein intake of high-quality sources is low among Asian Indians [6]. The increased prevalence of chronic diseases, especially T2D, has promoted initiatives to explore the role of protein intake on glucose and energy metabolism [21]. The key aspects explored as part of the present study were related to the protein consumption, i.e., the type and amount of protein. The data from the present study demonstrated that lower doses of WP and ISP were similar (i.e., insulinogenic), but at higher doses, WP had a greater insulinogenic response. The fact that, at the lower dose, both the protein types demonstrated similar response was promising, as this dose is translatable to clinical medicine/nutrition. For instance, the consumption of legume-containing foods may contribute to a lower incidence of postprandial hyperglycemia preventing complications associated with it including coronary heart diseases, atherosclerosis, T2D, and carcinogenesis [22]. The insulin-releasing capacity of WP and ISP could be attributed to their protein fraction [23]. The mechanism by which ISP could induce hyperinsulinemia is linked to higher amino acid alanine and arginine levels [9], stimulating the secretion of glucose-dependent insulinotropic polypeptide (GIP) [9]. The higher branched-chain amino acid (BCAA) levels in specific leucine concentration in WP could lead to a greater glucagon-like peptide-1 (GLP-1) response, which may, in turn, be responsible for the elevated insulin release [24]. However, the pathways by which ISP and WP might operate the insulinogenic action remain unknown. The lack of difference in insulinogenic response seen at a lower dose of protein in the present study needs further exploration. In a study on healthy individuals, the effects of casein, soy, and whey protein on various parameters including insulin response at different doses (10% and 25%) were studied [25]. The study demonstrated that, at a higher dose, insulin response was greater for whey than that for soy or casein. At a lower dose, insulin concentration increased more for casein than for soy or whey with no differences between whey and soy. Whey protein is considered a fast-absorbable protein resulting in greater aminoacidemia and a higher beta cell secretion than the ingestion of a slow absorbable protein like casein or soy protein [26]. Whether protein induced insulin response is dose dependent and there is a threshold at which the differential response starts emerging needs further exploration. The evidence towards the same could be seen in mouse islets models which demonstrated that insulin secretion depends on amino acid doses and glucose levels [27]. A recent systematic review and meta-analysis investigating the effect of replacing animal with plant protein on glycemic control among patients with diabetes observed that a higher plant protein intake resulted in better glycemic control [28]. However, the body of evidence comparing plant and animal protein intake on glycemic control and T2D risk has produced inconsistent results [29]. This is due to different types of commonly consumed plant proteins (e.g., soy, nut, seeds, beans, peas, and lentils) and animal proteins (e.g., meat, milk, fish, and eggs) that have been studied, each with their own set of protein quality characteristics and nonprotein components [30]. The current study, for the first time, explored the 2 most consumed protein sources of animal (whey) and plant (soy) protein and their impact on glucose homeostasis among Indian population. Legume (ISP) being an affordable protein could be a good alternative source, particularly in a setup where vegetarian sources might be acceptable culturally. The role of lean mass on glucose homeostasis affecting the cardiometabolic profile is well recognized; however, the current study did not study the impact of protein supplementation on lean mass nor strength, though the baseline DXA measurement was performed. Based on the systematic review by Lim et al., there seems to be a favorable effect on the lean mass in relation to animal protein compared to plant protein, and the benefit appears more pronounced among young individuals [31]. Meta-analyses performed in the same paper indicated that the protein source did not affect changes in strength. Similar findings were also reported by Messina et al., indicating that resistance exercise training (RET) when supplemented with whey or soy protein resulted in significant increases in muscle strength but found no difference between protein groups [32]. RET is a potent stimulus than protein supplementation for increasing muscle strength [33]. It will be worth exploring the impact of protein on muscle mass and strength and their association with glucose homeostasis, especially among Asian Indians. A randomized crossover clinical trial was performed among normal weight and normoglycemic subjects to assess the effect of the different proteins on second meal postprandial glycemia, and the data indicated that compared with control, whey and soy protein had a significant reduction in postprandial glycemia. However, the study did not explore the different protein doses or insulin responses [34]. Gunnerud et al. explored the efficacy of premeal bolus of whey and soy protein with or without added amino acids on glycemic, insulin, incretin, and amino acid response among healthy volunteers [35]. The premeal bolus displayed a lower glycemic response than the reference meal. However, there was no difference in the insulinemic responses between the meals. Data from the present study on the other hand demonstrated a significant insulin response to a higher dose (whey vs. soy protein). This could be due to the dose of protein used between the 2 studies (9 g vs. 15 and 30 g%). Kashima et al. studied soy protein isolate preload (20 g and 40 g) on glycemic control in young healthy subjects. The glycemic response for the soy protein isolate (40 g) was attributed to not only exaggerated insulin response but also to the noninsulin-dependent mechanism(s), i.e., gastric emptying [36]. The data from the present study demonstrated similar responses at higher doses and, in addition, compared soy and whey protein. A study among healthy women with similar macronutrient composition containing cod, milk, or soy protein indicated serum insulin response after the milk protein meal differed from that of the cod protein meal [37]. The insulin/glucose ratio for the cod protein meal was lower than that for the milk and soy protein meals. The use of food as a protein source depends on the protein fraction and can be highly variable [38]. In this context, though food as a source might be more feasible in the developing world where financial constraints might limit the use of protein as supplementation, more studies are required to compare the different food-based protein intervention and their impact on postprandial glycemia. The insulinogenic response to different doses of proteins is also relevant to the exogenous insulin dose, especially among individuals with type 1 diabetes [39].

The current study explored the thermogenic effect of WP and ISP at 2 doses (15% and 30%). Though oxygen consumption and energy expenditure increased following the protein meal, there was no difference in either dose or the type of protein. There were similar trends seen for CHO and fat oxidation as well. Acheson et al. demonstrated a significant thermogenic effect after a meal containing whey (50% protein) compared to casein and soy meals [11]. The dose used by Acheson et al. was high compared to that of the current study. The translation of such high-dose protein consumption is not feasible or recommended. Therefore, the current study explored a dose of protein that could be easily adapted in clinical practice. The lack of difference in the thermogenic effect in the present study could be due to the fact that it was performed in a relatively small homogenous population with the normal body weight. The impact of body composition especially body fat, including ectopic fat and muscle mass on the energy expenditure and substrate oxidation, following protein consumption needs to be further explored. This is of relevance as despite the normal body weight, it is proposed that Asian Indians have a greater predisposition to develop accumulation of body fat in ectopic sites [40, 41]. The protein intake could be one of the modes by which fat could be mobilized along with exercise. The present study only focused on healthy participants, and comparative data from obese or with type 2 diabetes could have added further value.

The current study did not demonstrate any changes in satiety. The questionnaire-based approach to evaluate satiety might not have uncovered subtle changes between the effects of proteins on satiety. The mechanisms by which protein may affect satiety remain elusive. Satiety involves a complicated interaction of psychological, behavioral, and physiological mechanisms [42]. It is proposed that the satiety centre could be sensitive to serum amino acid levels, and once the levels reach a certain point, hunger would cease [43, 44]. However, there is little evidence to support this. Another possible mechanism could be the relationship between satiety and incretin hormones [45]. With habitual low protein intake among Asian Indians, it will be interesting to explore the role of incretin hormonal changes and amino acid pool to understand the impact of protein on satiety among Asian Indians.

## 5. Conclusion

In conclusion, at lower doses, high-quality soy and whey did not elicit different insulinogenic responses, making both equally effective protein sources for the management of glucose metabolism when used in moderation. However, at higher doses, whey protein exhibited a greater insulinogenic response than soy protein. This provides important insight into Asian Indians who are at greater risk of developing T2D and may be seeking to increase protein intake.

## Figures and Tables

**Table 1:** Baseline characteristics of the study subjects (*n* = 15).

Age (yr)	22.20 ± 1.89
Weight (kg)	64.80 ± 8.64
Height (m)	1.71 ± 0.06
BMI (kg/m^2^)	22.02 ± 2.27
Fat percent (%)	24.31 ± 7.86
Fat (kg)	16.21 ± 6.28
Lean mass (kg)	46.33 ± 5.15
Physical activity level	1.18 ± 0.07

Data represented as mean ± SD; BMI-body mass index.

**Table 2:** Comparison of calorimetry measures and VAS scores between 4 intervention meals.

Calorimetry measures	15% soy	30% soy	15% whey	30% whey	*p* value
*VO*_*2*_ *(L/min)*					
iAUC	0.11 ± 0.02	0.12 ± 0.04	0.10 ± 0.04	0.14 ± 0.03	0.04
Peak response^b^	0.26 ± 0.01	0.27 ± 0.02	0.26 ± 0.02	0.27 ± 0.02	0.001

*VCO*_*2*_ *(L/min)*					
iAUC	0.16 ± 0.03	0.15 ± 0.04	0.13 ± 0.03	0.16 ± 0.03	0.18
Peak response	0.23 ± 0.02	0.24 ± 0.02	0.24 ± 0.02	0.24 ± 0.01	0.21

*RQ*					
iAUC	0.20 ± 0.12	0.21 ± 0.11	0.18 ± 0.11	0.18 ± 0.11	0.90
Peak response	0.94 ± 0.04	0.94 ± 0.05	0.93 ± 0.05	0.91 ± 0.04	0.26

*EE (kcal/min)*					
iAUC	2.60 ± 0.60	2.87 ± 0.77	2.50 ± 0.65	3.25 ± 0.51	0.06
Peak response^a,b^	5.32 ± 0.36	5.51 ± 0.41	5.43 ± 0.46	5.57 ± 0.46	0.001

*VAS scores*					
Hunger	4.42 ± 1.78	4.34 ± 1.83	4.66 ± 1.63	4.59 ± 1.40	0.89
Thought of food	4.23 ± 1.77	4.27 ± 1.74	4.55 ± 1.65	4.38 ± 1.41	0.76
Urge to eat	4.03 ± 1.28	4.29 ± 1.73	4.58 ± 1.56	4.39 ± 1.32	0.63
Fullness	3.87 ± 1.62	4.15 ± 1.68	3.90 ± 1.81	3.92 ± 1.62	0.95

Reported as mean ± SD; *p* values using RMANOVA. RQ, respiratory quotient; EE, energy expenditure; VAS, visual analogue scale. iAUC, incremental area under the curve. (a) 30% soy is significantly different from 15% soy and (b) 30% whey is significantly different from 15% whey.

## Data Availability

The data used to support the findings of this study are available from the corresponding author upon reasonable request.

## Supplementary Materials

Supplementary Materials Data on calorimetry measures, VO_2_, VCO_2_, RQ, and EE over the 5-hour measurement period are presented in the supplementary material. Supplementary Figure 1: calorimetric measures, VO_2_, VCO_2_, RQ, and EE between 4 intervention meals.
