# Supplementary material for: The Effect of Soy and Whey Protein Supplementation on Glucose Homeostasis in Healthy Normal Weight Asian Indians
Source: J Nutr Metab. 2023 Jul 10;2023:2622057. doi: 10.1155/2023/2622057 (PMC10352526; doi:10.1155/2023/2622057)
Supplement: Supplementary Materials — Data on calorimetry measures, VO2, VCO2, RQ, and EE over the 5-hour measurement period are presented in the supplementary material. Supplementary Figure 1: calorimetric measures, VO2, VCO2, RQ, and EE between 4 intervention meals. [file 2622057.f1.docx]

Supplementary Figure 1: Calorimetric measures, VO2, VCO2, RQ and EE between 4 intervention groups:

Group effect- 0.020,

Time effect-0.001,

Interaction effect -0.452
